# Antihyperglycemic and hypolipidemic effects of α, β-amyrin, a triterpenoid mixture from *Protium heptaphyllum* in mice

**DOI:** 10.1186/1476-511X-11-98

**Published:** 2012-08-06

**Authors:** Flávia Almeida Santos, Julyanne Torres Frota, Bruno Rodrigues Arruda, Tiago Sousa de Melo, Armenio André de Carvalho Almeida da Silva, Gerly Anne de Castro Brito, Mariana Helena Chaves, Vietla Satyanarayana Rao

**Affiliations:** 1 Department of Physiology and Pharmacology, Faculty of Medicine, Federal University of Ceará, Fortaleza, Ceará, Brazil; 2 Department of Clinical and Toxicological Analysis, Faculty of Pharmacy, Odontology and Nurse, Federal University of Ceará, Fortaleza, Ceará, Brazil; 3 Department of Chemistry, Federal University of Piauí, Teresina, Piauí, Brazil; 4 Department of Morphology, Faculty of Medicine, Federal University of Ceará, Fortaleza, Ceará, Brazil

**Keywords:** *Protium heptaphyllum*, α, β-amyrin, Pentacyclic triterpene, Antihyperglycemic and hypolipidemic effects, Mice

## Abstract

**Background:**

Pentacyclic triterpenes in general exert beneficial effects in metabolic disorders. This study investigated the effects of α, β-amyrin, a pentacyclic triterpene mixture from the resin of *Protium heptaphyllum* on blood sugar level and lipid profile in normal and streptozotocin (STZ)-induced diabetic mice, and in mice fed on a high-fat diet (HFD).

**Findings:**

Mice treated with α, β-amyrin (10, 30 and 100 mg/kg, p.o.) or glibenclamide (10 mg/kg, p.o.) had significantly reduced STZ-induced increases in blood glucose (BG), total cholesterol (TC) and serum triglycerides (TGs). Unlike glibenclamide that showed significant reductions in BG, TC and TGs in normoglycemic mice, α, β-amyrin did not lower normal blood sugar levels but at 100 mg/kg, manifested a hypolipidemic effect. Also, α, β-amyrin effectively reduced the elevated plasma glucose levels during the oral glucose tolerance test. Moreover, the plasma insulin level and histopathological analysis of pancreas revealed the beneficial effect of α, β-amyrin in the preservation of beta cell integrity. In mice treated orally with α, β-amyrin (10, 30 and 100 mg/kg) or fenofibrate (200 mg/kg), the HFD-associated rise in serum TC and TGs were significantly less. The hypocholesterolemic effect of α, β-amyrin appeared more prominent at 100 mg/kg with significant decreases in VLDL and LDL cholesterol and an elevation of HDL cholesterol. Besides, the atherogenic index was significantly reduced by α, β-amyrin.

**Conclusions:**

These findings reflect the potential antihyperglycemic and hypolipidemic effects of α, β-amyrin mixture and suggest that it could be a lead compound for drug development effective in diabetes and atherosclerosis.

## Introduction

Diabetes mellitus (DM) is a metabolic disorder characterized by hyperglycemia resulting from defects in insulin secretion, insulin action or both. Hyperglycemia and hyperlipidemia, as the most common features of diabetes mellitus, contribute to the development of microvascular and macrovascular complications of diabetes, which account for the morbidity and mortality of diabetes [1]. Search for compounds that normalize hyperglycemia, hyperlipidemia and ameliorate oxidative stress is an important objective in preventing diabetes-associated complications. None of the currently used medications reverse ongoing failure of beta cell function [2]. The search for newer drugs from natural sources, which are cost-effective and safe, without the long-term side effects may open new avenues for the treatment of diabetes and diabetes associated complications [3].

**Figure 1:**
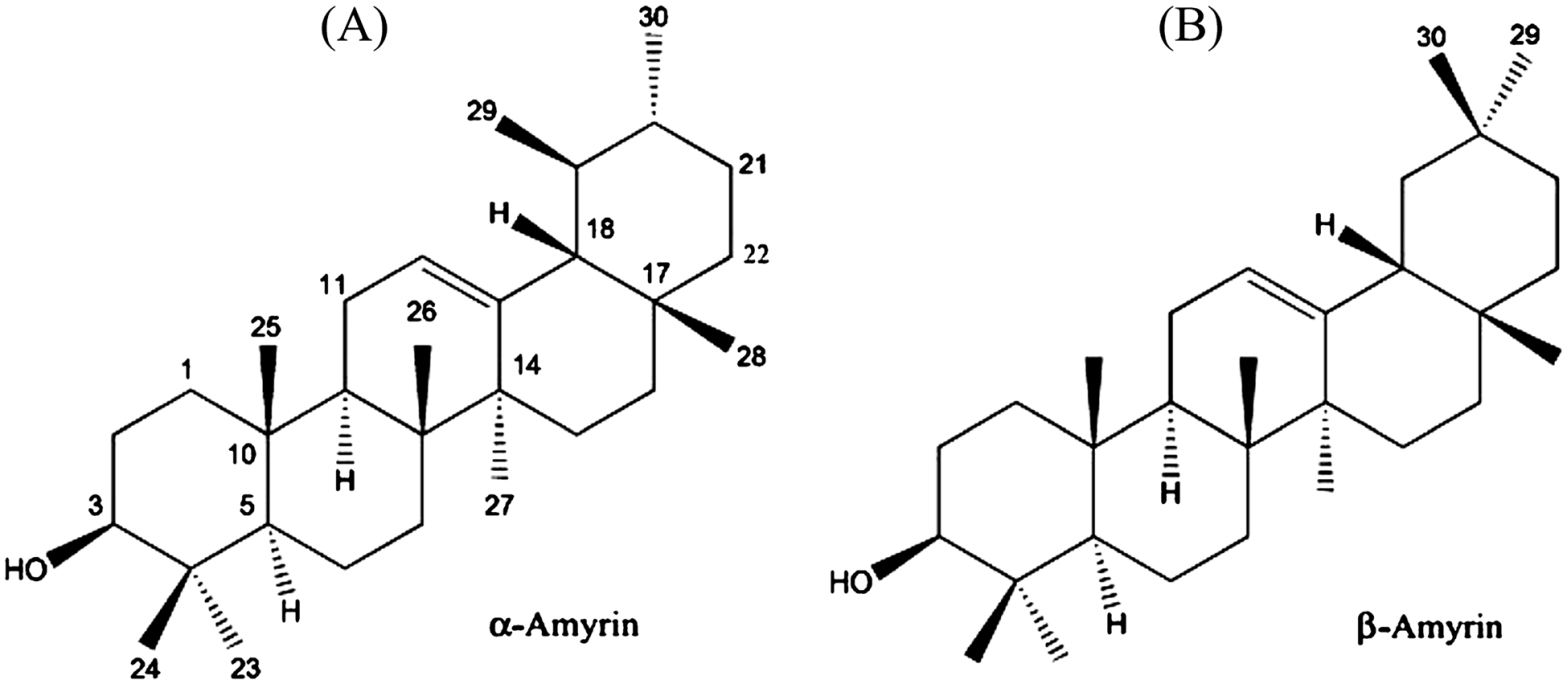
Chemical structures of α- amyrin (A) and β- amyrin (B).

In the recent past, many pentacyclic triterpenes were shown to improve lipoprotein lipase expression, insulin sensitivity and dyslipidemia [4-7]. The resin obtained from the trunk wood of *Protium heptaphyllum* (Aubl.) March (Burseraceae) is a reputed folk medicinal agent because of its analgesic and anti-inflammatory properties [8]. Chemical investigations have revealed the presence of α, β-amyrin, a pentacyclic triterpene as the major component of resin, and pharmacological studies have revealed anti-inflammatory and antinociceptive, antioxidant, antipruritic, gastroprotective and hepatoprotective effects, at non-toxic doses, which range from 10 to 100 mg/kg [9-16]. The pentacyclic triterpene α, β-amyrin (Figure 1) is constituted of triterpenes, that belongs to the group of ursane and oleanane series and that this class of agents has the chemical structure similar to that of a steroid and are extremely useful in prevention or treatment of many diseases in experimental animals, particularly those in which oxidative and inflammatory stress plays a key role in pathogenesis [17]. Several studies addressed on the molecular mechanism of the anti-inflammatory and antinociceptive actions of α, β-amyrin, using different experimental models. One study has shown its preventive or therapeutic anti-inflammatory potential in a murine model of trinitro-benzene-sulfonic acid (TNBS)-induced colitis, wherein α, β-amyrin was found to be as efficacious as dexamethasone in reversing the macroscopic and microscopic outcomes of TNBS-induced colitis through suppression of inflammatory cytokines and cyclooxygenase-2 levels, and by inhibition of NF-kB activation [11]. In other studies, α, β-amyrin ameliorated periodontal inflammation in rat model of ligature-induced periodontitis reducing the neutrophil infiltration, oxidative stress and the production of proinflammatory cytokine TNF- α, [10] and exhibited long-lasting antinociceptive and anti-inflammatory properties in 2 models of persistent nociception via activation of cannabinoid receptors and by inhibiting the production of cytokines and expression of NF-κB, and cyclooxygenase 2 [12,13]. Because both increased oxidative stress and augmented expression of inflammatory mediators are hallmarks of diabetes [18], and since α, β-amyrin demonstrates antioxidant and anti-inflammatory properties, we have examined this triterpene for its possible hypoglycemic effect on streptozotocin (STZ)-induced diabetic mice and lipid-lowering effect in mice fed on a high-fat diet. Although α, β-amyrin has previously been evaluated for hypoglycemic activity using the models of STZ-induced diabetic rat and diabetic db/db mice [19], to the best of our knowledge there were no such reports on α, β-amyrin and therefore the present study.

## Materials and methods

### Extraction and isolation of α, β-amyrin

The resinous exudates from the trunk wood of *Protium heptaphyllum* (March.) was collected from the municipal areas of Timon, Maranhão state of Brazil, after its identification by botanist Roseli Farias de Melo Barros. A voucher sample (#18247) has been deposited at the Herbarium Graziela Barroso of the Federal University of Piauí, Teresina, Brazil. The extraction and isolation of α, β-amyrin from the crude resin was carried out as described earlier [20] and its structural identity was confirmed by ^1^ H- and ^13^ C-NMR spectral analysis, based on the method developed by Gallegos and Roque [21] and comparison to literature data [22]. The ratio of α, β-amyrin in this mixture was 63:37.

### Animals

Male Swiss mice weighing 25–30 g were maintained at a constant room temperature (22–23°C) with light–dark cycles of 12 h and were allowed free access to water and standard laboratory chow. Experimental protocols were approved by the Institutional Committee on Care and Use of Animals for Experimentation (No. 22/09) in accordance with the guidelines of the National Institutes of Health, Bethseda, MD, U.S.A.

### Blood glucose and lipids in normal animals

Animals were divided into groups of 8 mice in each. A group served as normal control mice were treated orally with distilled water (10 mL/kg) only. The mice in the other groups received orally the vehicle (2% Tween 80 in distilled water, 10 mL/kg), α, β-amyrin (10, 30 or 100 mg/kg) or glibenclamide (10 mg/kg), once daily for 5 days. Following a 6 h fast, and at the end of the treatments on day-5, blood samples were drawn from the tail vein. Blood glucose, total cholesterol and triglycerides levels were determined using a semi-automatic analyser (Labquest, Labtest, Brazil) using diagnostic kits (Labtest, Brazil).

### Experimental induction of diabetes

The hypoglycemic activity of α, β-amyrin was evaluated in STZ-diabetic mice. Diabetes was induced by intraperitoneal injection of streptozotocin (STZ) (125 mg/kg in citrate buffer, pH 4.5) [23]. After 72 h of STZ administration, the mice with blood glucose level greater than 200 mg/dL were separated and divided into 6 groups of eight animals each. Group 1 was the normal control and contained mice treated orally with distilled water while group 2 was vehicle control and contained diabetic mice treated with vehicle (2% Tween 80 in distilled water, 10 mL/kg, p.o.). Mice in groups 3 to 6 were diabetic mice that received orally α, β-amyrin 10, 30 and 100 mg/kg and glibenclamide 10 mg/kg, respectively. Blood samples were collected from the tail vein at 3 h and 5 h after the treatments and the blood glucose was analysed by colorimetric method in a semi-automatic analyser (Labquest, Labtest, Brasil) using diagnostic kit (Labtest, Brazil). Plasma insulin levels were measured from 5 h blood sample using a rat/mouse insulin enzyme-linked immunosorbent assay according to the manufacturer’s instructions. The animals were euthanized; pancreas collected, fixed in 10% formalin, and were then embedded in paraffin to prepare sections using standard protocols. Pancreatic sections were stained with Hematoxylin and Eosin and were examined under light microscopy for morphological analysis.

### Oral Glucose Tolerance Test (OGTT)

Mice were divided into groups of 08 mice each. A group served as normal control mice treated orally with distilled water only. The mice in the other groups received vehicle (2% Tween in distillated water, 10 mL/kg, p.o.), α, β-amyrin (10, 30 or 100 mg/kg, p.o.) or glibenclamide (10 mg/kg, p.o.). Vehicle, α, β-amyrin or glibenclamide were administered once daily for five days. On day 5 after a 6 h starvage, and 2 h after the respective treatments, blood samples from the tail vein were collected and the blood glucose was determined (0 h). Groups 2–6 were administered glucose (2 g/kg) orally as a bolus. Blood samples of all groups were collected from tail vein at 30, 60 and 90 min time point for measurment of blood glucose.

### Hypercholesterolaemia induced by diet

Albino male mice were divided into groups of eight in each. One group served as normal control and received normal pelleted feed and had free access to drinking water. The mice in the other groups received, in addition to pelleted diet and water, a hyperlipidemic diet witch was a combination of sunflower oil (10 mL/kg), 5% cholesterol and 0.5% cholic acid by oral gavage for two weeks [24]. Vehicle (2% Tween 80 in distilled water, 10 mL/kg), α, β-amyrin (10, 30 or 100 mg/kg) or fenofibrate (200 mg/kg) were administered orally once daily to mice 4 h after feeding. The body weight of animals was recorded at the end of the second week. At the end of two weeks, following an overnight fast, blood samples from the tail vein were collected and serum total cholesterol (TC), HDL-cholesterol (HDL-c) and triglycerides (TG) were analysed by colorimetric method in a semi-automatic analyzer (Labquest, Labtest, Brazil) using diagnostic kits (Labtest, Brazil). The serum low-density lipoprotein-cholesterol (LDL-c) and very-low-density lipoprotein-cholesterol (VLDL-c) concentrations were calculated using the Friedewald formula [20], where LDL-c = TC – (HDL-c + VLDL-c) and VLDL-c = TG/5. The atherogenic index (AI) was expressed as LDL-c + VLDL-c/HDL-c.

### Statistical analysis

All data are presented as mean ± SEM. The data were evaluated by one-way analysis of variance with Newman-Keuls posthoc test using the GraphPad Prism 4.0 statistical program. The level of significance was set at p ≤ 0.05.

## Results

Table 1 shows that a 5-day administration of α, β-amyrin (10, 30 and 100 mg/kg) in normal mice did not significantly reduce the blood glucose level. However, glibenclamide (10 mg/kg) caused a significant reduction in blood glucose level when compared to vehicle-treated control group. Treatments with α, β-amyrin (100 mg/kg) as well as glibenclamide also showed significantly reduced total cholesterol and triglycerides levels.

**Table 1:** Effects of α, β-amyrin and glibenclamide treatments on blood glucose, total cholesterol and triglycerides in normal mice

**Group**	**Dose (mg/kg)**	**Blood glucose (mg/dL)**	**Total Cholesterol (mg/dL)**	**Triglycerides (mg/dL)**
Normal Control	**-**	114.50 ± 5.11	93.60 ± 11.66	143.10 ± 11.40
Vehicle Control	**-**	115.75 ± 5.61	96.00 ± 5.12	151.10 ± 14.62
α,β-amyrin	10 mg/kg	106.83 ± 1.47	97.63 ± 10.39	114.60 ± 8.19
α,β-amyrin	30 mg/kg	101.67 ± 2.55	92.75 ± 7.74	116.00 ± 8.01
α,β-amyrin	100 mg/kg	100.43 ± 4.92	72.57 ± 9.32^a,b^	97.00 ± 9.76^a,b^
Glibenclamide	10 mg/kg	87.14 ± 5.21^a,b^	69.17 ± 2.18^a,b^	82.38 ± 2.66 ^a,b^

Each value represents the mean ± SEM, *n* = 8 mice. ^a^ p < 0.05 compared with normal control. ^b^ p < 0.05 compared with vehicle control.

**Figure 2:**
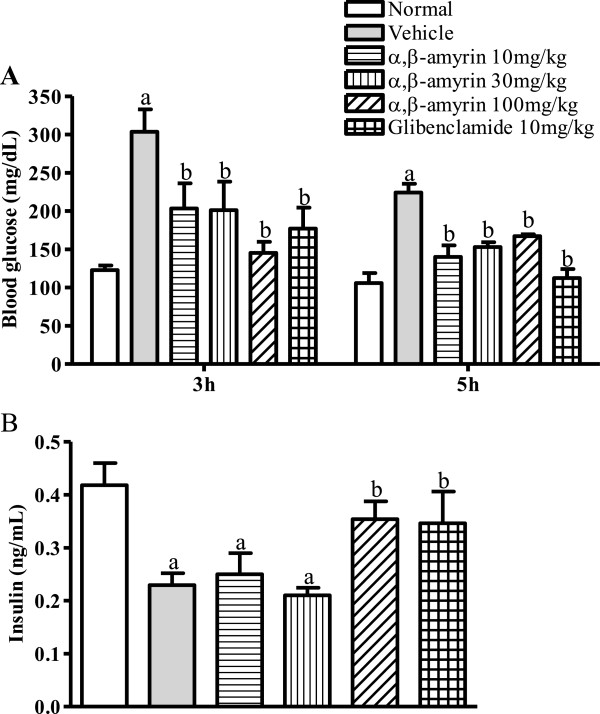
**Treatment effects of α, β-amyrin or glibenclamide on blood glucose levels and insulin levels in streptozotocin (STZ)-induced diabetic mice.** Each bar represents the mean ± SEM (n *=* 8) of blood glucose (mg/dL) at time 3 h and 5 h (**A**) or insulin at time 5 h (**B**). ^a^ p < 0.05 compared with normal control. ^b^ p < 0.05 compared with vehicle control.

Figure 2 shows the effect of α, β-amyrin and glibenclamide in streptozotocin-diabetic mice. In vehicle control group the blood glucose level was significantly elevated 3 h and 5 h after treatment when compared with normal control mice. The triterpenoid (10, 30 and 100 mg/kg) and glibenclamide (10 mg/kg) treatments were able to reduce significantly the STZ-associated increase in blood glucose level at time points of 3 h and 5 h. Both α, β-amyrin (100 mg/kg) and glibenclamide (10 mg/kg) were able to elevate significantly the insulin levels at time 5 h.

**Figure 3:**
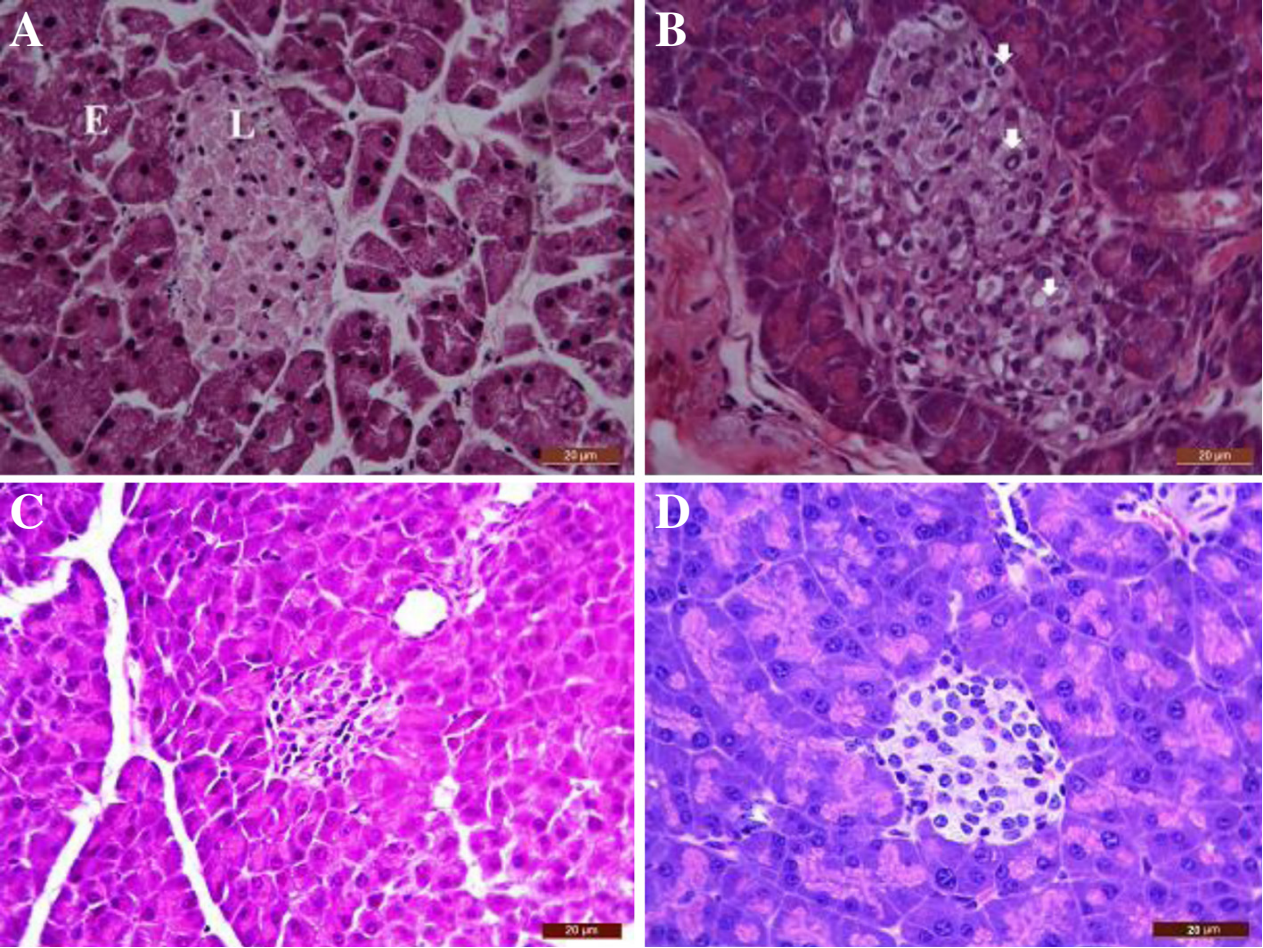
**Histology of mouse pancreas stained by Hematoxylin & Eosin.** Normal islets of Langerhans (L) surrounded by exocrine portion (E) of pancreatic tissues in control mice (**A**). Destruction and distortion of endocrine cells in STZ-diabetic mice with the presence of necrotic cells and inflammatory cells (arrowhead) (**B**). **C**: Improvement in morphology of islets of Langerhans with α, β-amyrin (100 mg/kg) pretreatment in STZ-diabetic mice; **D**: Islets of Langerhans of diabetic mice treated with glibenclamide (10 mg/kg) showing restoration of normal cellular population. (H & E 400×), Bar: 20 μm.

Figure 3 depicts the results of histological analysis of pancreas from normal or STZ-diabetic mice treated or not with either α, β-amyrin or glibenclamide. Figure 3A representing pancreatic section from normal mice that show well defined islets of Langerhans surrounded by exocrine portion of pancreatic tissues. Pancreas sections from untreated diabetic mice (Figure 3B) showed both destruction and distortion of endocrine cells. Apart from this, necrotic cells and inflammatory cells were present. The diabetic mice treated with α, β-amyrin (100 mg/kg) showed no morphological changes of islets of Langerhans and the features were almost similar to control pancreas (Figure 3C). Histological sections from STZ-diabetic mice treated with glibenclamide also showed protection from the destructive effect of STZ, showing normal cellular population (Figure 3D)**.**

**Figure 4:**
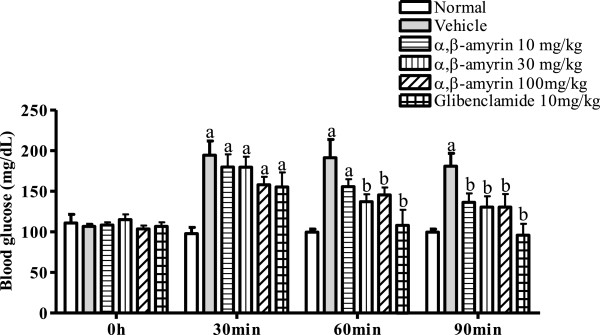
**Treatment effects of α, β-amyrin and glibenclamide in oral glucose tolerance test.** Each bar represents the mean ± SEM (n *=* 8). ^a^ p < 0.05 compared with normal control. ^b^*p* < 0.05 compared with vehicle control.

Figure 4 depicts the pretreatment effects of α, β-amyrin (10, 30 and 100 mg/kg) and glibenclamide (10 mg/kg) on oral glucose tolerance test. Before the glucose load, the basal blood glucose levels were not significantly different between the groups. In this test, while normal control showed no significant changes in the levels of blood glucose, at all time points (0, 30, 60, and 90 min) of observation, vehicle-treated mice loaded with oral glucose manifested an increase in blood glucose at the time points of 30, 60 and 90 min. The five-day oral pretreatment with α, β-amyrin (10, 30 and 100 mg/kg) and glibenclamide significantly improved the glucose tolerance at the 30, 60 and 90 min periods.

The serum lipid profiles of mice after a two weeks normal or high-fat diet are shown in Table 2. The normal group of mice fed on normal chow diet had serum total cholesterol and triglycerides levels of 119.00 ± 5.86 and 48.85 ± 5.94 mg/dL, respectively as against 225.40 ± 10.93 and 258.80 ± 27.48 mg/dL, respectively, in mice on the high-fat diet. Orally administered α, β-amyrin (10, 30 and 100 mg/kg) and fenofibrate (200 mg/kg) significantly decreased the high-fat-diet-induced increases in total cholesterol and triglycerides. The cholesterol fractions HDL, VLDL and LDL were significantly increased by a high-fat diet. α, β-amyrin (10, 30 and 100 mg/kg) and fenofibrate (200 mg/kg) significantly reduced the LDL levels and only α, β-amyrin at doses of 30 and 100 mg/kg reduced the VLDL levels. α, β-amyrin (30 and 100 mg/kg) and fenofibrate significantly increased the HDL levels. α, β-amyrin (10, 30 and 100 mg/kg) and fenofibrate significantly lowered the atherogenic index. At the end of two weeks, significant differences were observed in the final body weights between the normal diet control group (30.63 ± 1.62 g) and the high-fat diet control (37.75 ± 0.56 g) mice. When compared to vehicle-treated high-fat diet mice, the observed final body weights were significantly lower in animal groups that received α, β-amyrin 10, 30 and 100 mg/kg (32.25 ± 1.72; 32.25 ± 0.75 and 30.63 ± 0.94 g, respectively) and fenofibrate 200 mg/kg (31.14 ± 1.78 g).

**Table 2:** Effects of α, β-amyrin and fenofibrate treatments on serum lipid parameters in mice

**Lipid parameter**	**Normal diet (control)**	**High-fat diet and treated**				
		**Vehicle Control**	**α, β-amyrin 10 mg/kg**	**α, β-amyrin 30 mg/kg**	**α, β-amyrin 100 mg/kg**	**Fenofibrate 200 mg/kg**
Total cholesterol (mg/dL)	119.00 ± 5.86	225.40 ± 10.92^a^	175.90 ± 9.62^b^	175.60 ± 13.32^b^	166.30 ± 8.97^b^	143.40 ± 7.48^b^
Triglycerides (mg/dL)	48.85 ± 5.94	258.80 ± 27.48^a^	204.7 ± 26.01^b^	146.70 ± 21.24^b^	50.50 ± 6.66^b^	70.71 ± 13.48^b^
HDL-c (mg/dL)	26.13 ± 1.42	39.00 ± 1.59^a^	48.63 ± 4.78	63.38 ± 7.12^b^	71.25 ± 3.21^b^	71.14 ± 3.92^b^
VLDL-c (mg/dL)	9.75 ± 1.18	48.91 ± 5.45^a^	40.94 ± 5.20	33.65 ± 4.21^b^	10.10 ± 1.33^b^	14.14 ± 2.69^b^
LDL-c (mg/dL)	83.29 ± 7.07	137.20 ± 12.32^a^	81.38 ± 16.00^b^	100.10 ± 12.88^b^	90.65 ± 11.22^b^	58.14 ± 8.62^b^
Atherogenic index (AI)	3.65 ± 0.32	4.78 ± 0.34^a^	3.01 ± 0.45^b^	2.23 ± 0.49^b^	1.46 ± 0.22^b^	1.09 ± 0.21^b^

Each value represents the mean ± S.E.M. (n = 8). ^a^ p < 0.05 compared with normal diet-fed control. ^b^ p < 0.05 compared with vehicle control.

## Discussion

The results of this study clearly demonstrates that the pentacyclic triterpene α, β-amyrin isolated from *Protium heptaphyllum* exerts antihyperglycemic and hypolipidemic effects in mouse models of STZ-induced diabetes and high-fat diet-induced hyperlipidemia, highly relevant to experimental diabetes research [25-27]. These results are consistent with our previous reports that show similar effects with other pentacyclic triterpenes like betulinic, oleonolic, and ursolic acids in yet another mouse model of high-fat fed-induced obesity [28-30]. Excessive production of nitric oxide and the subsequent increase in local oxidative stress, or altered intracellular Ca^+2^ regulation, are some suggested pathogenic mechanisms in pancreatic beta-cell death and the development of insulin-dependent diabetes mellitus-induced by STZ [31]. Many conventional drugs used to treat diabetes act by improving insulin sensitivity, increasing insulin production, and or by decreasing the level of blood glucose. Decrease in blood glucose might be a consequence of reduced glucose absorption from gut, inhibition of glucose production in hepatic tissue, and or increased uptake by muscle and adipose tissue. The results of the OGTT further support these findings.

The molecular mechanism(s) by which α, β-amyrin manifests antihyperglycemic and antilipidemic effect are not clear. A recent study has largely explored the molecular mechanisms through which α, β-amyrin exerts its anti-inflammatory and anitinoceptive actions, including the first demonstration that this pentacyclic triterpene interacts with cannabinoid system [12]. An yet another study reported that α, β-amyrin has an inhibitory effect on 2-arachidonoylglycerol (2-AG) hydrolysis, and consequently increases the endocannabinoids [13]. Animal studies clarified the important role of endocannabinoid system and the cannabinoid 1 (CB1) receptors in the hypothalamus and in the limbic system in mediating orexigenic effects [32]. Recent studies indicate that some CB1 ligands may directly bind and allosterically regulate Kir6.2/SUR1 K(ATP) channels like other potassium channel openers (KCOs) and thus cause body weight-independent improvements in insulinemia and glycemia [33,34]. We hypothesise that α, β-amyrin improves glycemia possibly by its interaction with cannabinoid system and warrants a future investigation.

Triterpenoids in general have poor bioavailability and so is the case with α, β-amyrin. Pharmacokinetic investigations with a reliable GC-MS method reveal that amyrin has low bioavailabilty but a long elimination half life, following its oral administration to normal rats [35]. However, earlier works [9-16] show that it is orally effective in several animal models of inflammation, nociception, gastroprotection and hepatoprotection. Also, the present study demonstrates the oral efficacy of alpha, beta-amyrin in reducing hyperglycemia and hyperlipidemia in the experimental models of STZ-induced diabetes and diet induced hyperlipidemia.

>A pathogenic role for tumor necrosis factor-alpha (TNF-α) in mice with Type 1- and 2- diabetes and the efficacy of anti- TNF-α treatment in ameliorating hyperglycemia and restoring normal insulin has been recently addressed [36]. In a previous study, we demonstrated the anti-inflammatory and antioxidant effects of α, β-Amyrin in rodent models of cerulein-induced acute pancreatitis and ligature-induced acute periodontitis [9,10], wherein this triterpenoid was found to significantly inhibit the serum level of pro-inflammatory cytokine TNF- α as well as the increases in myeloperoxidase (MPO) activity and and thiobarbituric acid-reactive substances (TBARS). In the present study, treatment with α, β-amyrin effectively restored the reduced insulin levels seen in streptozotocin-diabetic controls. These observations strongly suggest that α, β-amyrin by its anti-inflammatory and anti-oxidative effects, may be in part, exercise beneficial effects in diabetic rats.

It is known that glibenclamide binds to K_ATP_ + channel on the cell membrane of pancreatic beta cells thereby blocking out flux of potassium ions and opening voltage gated Ca_2_+ channels. This rise in intracellular calcium promoted by glibenclamide eventually leads to increased secretion of insulin [37]. Glibenclamide used as a positive control in the study is a sulfonylurea derivative clinically used for treating hyperglycemia but has been associated with severe and sometimes fatal hypoglycemia [38]. Unlike sulphonylurea compound glibenclamide, oral administration of α, β-amyrin failed to produce hypoglycemia in normal animals. This suggests that the mode of action of the plant terpenoid is probably mediated by other mechanism(s). Literature reports suggest that many plant-derived triterpenoids might enhance glucose uptake by acting as insulin mimics and as insulin sensitizers [39], some exhibit alpha-glucosidase inhibition [40]. Insulin resistance affects not only the regulation of carbohydrate metabolism but all aspects of lipid and lipoprotein metabolism and is associated with elevated VLDL and increased triglycerides [41]. Therefore the observed diminution of VLDL and the hypertriglyceridemia by α, β-amyrin might be a result of improved insulin sensitivity.

In summary, we have demonstrated that α, β-amyrin, a pentacyclic triterpene ameliorates hyperglycemia and dyslipidemia, reduces atherogenic risk factor, and improves glucose tolerance in mice possibly by its anti-inflammatory and antioxidant effects. However, the molecular mechanism(s) underlying these effects of α, β-amyrin remains to be established.

## Abbreviations

AI: Atherogenic index; 2-AG: 2-arachidonoylglycerol; BG: Blood glucose; CB1: Cannabinoid 1; DM: Diabetes mellitus; HDL-c: High density lipoprotein-cholesterol; KCOs: Potassium channel openers; LDL-c: Low density lipoprotein-cholesterol; MPO: Myeloperoxidase; OGTT: Oral glucose tolerance test; STZ: Streptozotocin; TBARS: Thiobarbituric acid-reactive substances; TC: Total cholesterol; TG: Triglycerides; TNF-α: tumor necrosis factor-alpha; VLDL-c: Very low density lipoprotein-cholesterol.

## Competing interests

The authors declare that they have no competing interests.

## Author’s contributions

JTF, TSM, BRA, NTPC performed animal experiments and statistical analysis, and contributed to writing of the manuscript; MHC, AACAS contributed to isolation and chemical identification of α, β-amyrin; GACB performed the histological analysis; VSR and FAS participated in the design and coordination of the study and contributed to writing of the manuscript. All authors read and approved the final version of the manuscript.
